# Complete Genome Sequence of a Novel Aquareovirus That Infects the Endangered Fountain Darter, *Etheostoma fonticola*

**DOI:** 10.1128/genomeA.01405-16

**Published:** 2016-12-22

**Authors:** Luke R. Iwanowicz, Deborah D. Iwanowicz, Cynthia R. Adams, Teresa D. Lewis, Thomas M. Brandt, Robert S. Cornman, Lakyn Sanders

**Affiliations:** a U.S. Geological Survey, Leetown Science Center, Kearneysville, West Virginia, USA; b U.S. Fish and Wildlife Service, Midwest Fisheries Center, Onalaska, Wisconsin, USA; c U.S. Fish and Wildlife Service, San Marcos Aquatic Resources Center, San Marcos, Texas, USA; d U.S. Geological Survey, Fort Collins Science Center, Fort Collins, Colorado, USA

## Abstract

Here, we report the complete genome of a novel aquareovirus isolated from clinically normal fountain darters, *Etheostoma fonticola*, inhabiting the San Marcos River, Texas, USA. The complete genome consists of 23,958 bp consisting of 11 segments that range from 783 bp (S11) to 3,866 bp (S1).

## GENOME ANNOUNCEMENT

The fountain darter, *Etheostoma fonticola*, is a small benthic fish endemic to the headwaters of the San Marcos and Comal Rivers of central Texas, USA (1). This fish is currently listed as federally endangered, and the current recovery plan requires biological research necessary for monitoring, management, and restoration (2–4). During 2003, an unknown viral agent was isolated from a pool of clinically normal fountain darters collected from the San Marcos River. It is unknown if this virus is a pathogen of concern. Identification of this virus was a critical component of disease management for this species. Here, we present the complete genome sequence of this novel aquareovirus.

Aquareoviruses are nonenveloped icosahedral virions that contain a genome composed of 11 segments of double-stranded RNA that code for 12 proteins. At present, seven species of *Aquareovirus* are recognized by the International Committee on Taxonomy of Viruses based on nucleotide or amino acid identity of segment 2 or segment 10, and from the results of RNA-RNA hybridization experiments (5, 6). For decades, aquareoviruses have been isolated from moribund and clinically normal fish and shellfish (7, 8). The pathogenicity of aquareoviruses ranges from apathogenic to highly virulent. Pathogenic aquareoviruses are most commonly observed in fishes maintained for aquaculture (9).

Ribosomal RNA was depleted from total RNA isolated from infected CHSE-214 cells using a Ribo-Zero kit. High-throughput sequencing was performed using a combination of Ion Torrent PGM and Illumina MiSeq platforms. Genome assembly was conducted using CLC Genomics Workbench (version 7.5) and CAP3 (10). Dideoxy sequencing was used to close sequence gaps. The composite average mapping coverage of each segment ranged from 241 to 9,133×. Blastx queries of all segments in the NCBI database best matched aquareoviruses that infect fish but clearly identified this virus as unique. We confirmed full-length sequences via the identification of the conserved 5′- and 3′-terminal sequences (GUUU and CAUC, respectively). The complete genome of this virus is 23,958 nucleotide bp, and the size of the segments are as follows S1, 3,947 bp; S2, 3,866 bp; S3, 3,687 bp; S4, 2,554 bp; S5, 2,242 bp; S6, 2,057 bp; S7, 1,317 bp; S8, 1,317 bp; S9, 1,118 bp; S10, 987 bp; and S11, 783 bp. The G+C content was 56.6%, which is consistent with that of other aquareoviruses (11). A single open reading frame (ORF) was identified in each segment, with the exception that S7 contained two partially overlapping ORFs. A total of 12 ORFs were identified that encoded putative structural and nonstructural proteins typical of aquareoviruses. Here, we name this novel virus *Etheostoma fonticola aquareovirus* (EFAReV). The public availability of this complete genome sequence will facilitate the development of diagnostic molecular markers.

### Accession number(s).

The genome sequence of the *Etheostoma fonticola* aquareovirus was submitted to GenBank with the accession numbers KU194213 to KU194223 that correspond to genomic segments 1 to 11, respectively.

## References

[1] Hubbs C, Edwards RJ, Garrett GP 2008 An annotated checklist of the freshwater fishes of Texas, with keys to identification of species, 2nd ed. Texas Academy of Science, Edinburg, TX http://hdl.handle.net/2152/6290.

[2] Bonner TH, McDonald DL 2005 Threatened fishes of the world: *Etheostoma fonticola* (Jordan & Gilbert 1886) (Percidae). Environ Biol Fishes 73:333–334. doi:10.1007/s10641-004-4132-6.

[3] Brandt TM, Graves KG, Berkhouse CS, Simon TP, Whiteside BG 1993 Laboratory spawning and rearing of the endangered fountain darter. Prog Fish Cult 55:149–156. doi:10.1577/1548-8640(1993)055<0149:LSAROT>2.3.CO;2.

[4] Olsen JB, Kinziger AP, Wenburg JK, Lewis CJ, Phillips CT, Ostrand KG 2016 Genetic diversity and divergence in the fountain darter (*Etheostoma fonticola*): implications for conservation of an endangered species. Conserv Genet 17:1393–1404. doi:10.1007/s10592-016-0869-7.

[5] King AM, Lefkowitz E, Adams MJ, Carstens EB 2011, Virus taxonomy: ninth report of the International Committee on Taxonomy of Viruses. Academic Press, London, United Kingdom.

[6] Rangel AAC, Rockemann DD, Hetrick FM, Samal SK 1999 Identification of grass carp haemorrhage virus as a new genogroup of aquareovirus. J Gen Virol 80:2399–2402. doi:10.1099/0022-1317-80-9-2399. 10501493

[7] Lupiani B, Subramanian K, Samal SK 1995 Aquareoviruses. Annu Rev Fish Dis 5:175–208. doi:10.1016/0959-8030(95)00006-2.

[8] Crane MSJ, Carlile G 2009 Aquareoviruses. *In* Mahy BWJ, Van Regenmortel MHV (ed), Desk encyclopedia of animal and bacterial virology, 1st ed. Academic Press, London, United Kingdom.

[9] Blindheim S, Nylund A, Watanabe K, Plarre H, Erstad B, Nylund S 2015 A new aquareovirus causing high mortality in farmed Atlantic halibut fry in Norway. Arch Virol 160:91–102. doi:10.1007/s00705-014-2235-8. 25348270 PMC4284399

[10] Huang X, Madan A 1999 CAP3: a DNA sequence assembly program. Genome Res 9:868–877. doi:10.1101/gr.9.9.868. 10508846 PMC310812

[11] Attoui H, Fang Q, Mohd Jaafar F, Cantaloube JF, Biagini P, de Micco P, de Lamballerie X 2002 Common evolutionary origin of aquareoviruses and orthoreoviruses revealed by genome characterization of Golden shiner reovirus, Grass carp reovirus, Striped bass reovirus and golden ide reovirus (genus *Aquareovirus*, family Reoviridae). J Gen Virol 83:1941–1951. 12124458 10.1099/0022-1317-83-8-1941

